# Prognostic and predictive impact of gene expression in node‐positive early breast cancer patients receiving dose‐dense versus standard‐dose adjuvant chemotherapy

**DOI:** 10.1002/1878-0261.13435

**Published:** 2023-04-24

**Authors:** Mattea Reinisch, Simona Bruzas, Oleg Gluz, Beyhan Ataseven, Peter Schmid, Javier Cortés, Jens‐Uwe Blohmer, Satyendra Shenoy, Mark H. Dyson, Christine Dittmer‐Grabowski, Ouafaa Chiari, Hakima Harrach, Daniel Gebauer, Alexander Traut, Sherko Kuemmel

**Affiliations:** ^1^ Breast Unit Kliniken Essen‐Mitte Germany; ^2^ Bethesda Hospital Breast Center Niederrhein Mönchengladbach Germany; ^3^ Department of Gynecology and Gynecologic Oncology Kliniken Essen‐Mitte Germany; ^4^ Department of Obstetrics and Gynecology University Hospital, LMU Munich Germany; ^5^ Centre for Experimental Cancer Medicine, Barts Cancer Institute Queen Mary University of London UK; ^6^ International Breast Cancer Centre (IBCC) Quiron Group Barcelona Spain; ^7^ Vall d'Hebron Institute of Oncology (VHIO) Barcelona Spain; ^8^ Department of Gynecology with Breast Center Charité‐Universitätsmedizin Berlin, corporate member of Freie Universität Berlin and Humboldt‐Universität zu Berlin Germany; ^9^ Institute for Pathology Viersen Germany

**Keywords:** adjuvant chemotherapy, dose‐dense chemotherapy, early breast cancer, gene signature, lymph node‐positive

## Abstract

The utility of multigene expression assays in advanced (≥ 4 positive lymph nodes) early breast cancer (EBC) is limited. We conducted exploratory transcriptomic analysis of 758 genes (Breast Cancer 360 panel, nCounter^®^ platform; NanoString) in primary tumor samples collected during a phase 3 trial comparing adjuvant taxane‐containing dose‐dense chemotherapy (ddCTX) versus standard‐dosed chemotherapy (stCTX) in resected EBC with ≥ 4 positive lymph nodes. Prognostic and predictive associations with disease‐free survival (DFS) and overall survival (OS) were evaluated by Cox regression with false discovery rate (FDR) adjustment. Data were available from tumor samples of 141/226 patients (median follow‐up: 14 years). Several genes/signatures, including immune markers, showed prognostic relevance in unadjusted analyses. Of these, two remained significant after multiplicity adjustment: a positive effect on DFS of programmed cell death 1 ligand‐2 (PD‐L2) in the ddCTX arm (univariate HR: 0.53, FDR‐adjusted *P* = 0.036) and a negative effect on OS of HER2‐enriched (HER2‐E) signature in the stCTX arm (univariate HR: 5.40, FDR‐adjusted *P* = 0.036). Predictive analyses showed greater DFS benefit of ddCTX in tumors with high antigen processing machinery (APM) expression (multivariate interaction *P* = 0.024). Multigene expression assays have a prognostic and predictive potential in advanced EBC, and further investigation is warranted in order to identify candidates for de‐escalated treatment. In addition, intrinsic subtype and immune gene expression have predictive potential.

## Graphical abstract

Early breast cancer patients with advanced nodal involvement have a varied course of disease, thereby constituting a clinically high‐risk subgroup. High‐throughput transcriptomic analyses provide a snapshot of gene expression patterns in tumors. This strategy has the potential to identify prognostic and predictive genes and gene signatures, which could further guide in the selection of appropriate treatment regimens.

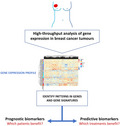

**Abbreviations**
APM: antigen processing machinery
AR: androgen receptor
BC360: Breast Cancer 360™
CI: confidence interval
CMF: cyclophosphamide, methotrexate, and 5‐fluorouracil
ddCTX: dose‐dense chemotherapy
DFS: disease‐free survival
EBC: early breast cancer
ER: estrogen receptor
ESR1: estrogen receptor 1
FDR: false discovery rate
HER2: human epidermal growth factor receptor 2
HER2‐E: HER2‐enriched
HR: hazard ratio
IDO1: indoleamine 2,3‐dioxygenase‐1
IEC: institutional ethics committee
mRNA: messenger ribonucleic acid
OS: overall survival
PD‐L1: programmed cell death 1 ligand‐1
PD‐1: programmed cell death protein 1
PD‐L2: programmed cell death 1 ligand‐2
PFR: progesterone receptor
ROR: risk of recurrence
stCTX: standard‐dosed chemotherapy
TIGIT: T‐cell immunoreceptor with immunoglobulin and immunoreceptor tyrosine‐based inhibition motif domain
TIL: tumor‐infiltrating lymphocytes
TIS: tumor inflammation signature

## 1 Introduction

In early breast cancer (EBC), anthracycline‐ and taxane‐containing adjuvant chemotherapy (CTX) reduces recurrence and breast cancer‐specific mortality [1]. Incremental benefits are obtained with the biweekly dose‐dense chemotherapy (ddCTX) regimens in comparison with the triweekly standard dosing [2]. Despite the well‐recognized heterogeneity of breast cancer, the proportional gains with CTX (versus no CTX) and ddCTX (versus standard‐dosed chemotherapy [stCTX]) are mostly independent of classic clinicopathologic factors [1, 2]. Neo/Adjuvant chemotherapy use is therefore largely based on the absolute risk, which reflects the expected absolute benefit, within each clinical subgroup [3]. However, it remains challenging to identify which individual patients will benefit, either from chemotherapy in general or from dose‐dense regimens, and the risk of under‐ or overtreatment remains considerable, especially in luminal EBC despite introduction of modern multigene expression assays. Hence, the development of biomarkers to further understand the heterogeneity of breast cancer and to reduce overtreatment through rational de‐escalation of therapy for selected patients is of clinical priority.

Significant insights into the diverseness of breast cancer have been provided by multigene expression assays that generate a risk score based on the transcriptomic characteristics of the tumor [4]. However, it is still open to debate whether *post hoc* analyses of recent large phase 3 trials such as MINDACT [5], TAILORx [6], ADAPT [7], and RxPONDER [8] have demonstrated predictive relevance for selected multigene parameters with respect to adjuvant chemotherapy. The only trial to formally test interaction between chemotherapy and signature‐derived risk score—RxPONDER—failed to detect any significant interaction. Limitations in the other trials such as protocol deviations, suboptimal regimens, low event rates, and some such also preclude making a definite conclusion on the utility of multigene expression assays as prognostic or predictive tools for identifying survival benefits. It is therefore sensible to consider this caveat before indicating that patients with 0–3 involved axillary nodes and low transcriptomic risk can forgo chemotherapy without compromising outcomes.

Patients with more advanced nodal involvement (≥ 4 positive nodes) generally receive a recommendation for chemotherapy, including dose‐dense regimens [9], but the course of disease varies widely even in this clinically high‐risk subgroup. To date, the prognostic and predictive potential of multigene assays has been insufficiently studied in this population.

In order to answer this question, we attempted to identify whether there was an association between treatment/survival outcomes and the expression of genes and gene signatures relevant to breast cancer in tumor samples collected during the course of a randomized phase 3 trial for which long‐term follow‐up was available [10, 11]. We also explored prognostic/predictive associations within the intrinsic group subtypes who, due to the period of enrolment (1996–2000), did not receive HER2‐targeted therapy or platinum agents.

## 2 Materials and methods

### 2.1 Study design

We conducted a transcriptomic analysis of primary tumors collected from patients enrolled in a randomized, open‐label, multicenter, phase 3 trial, which has been reported previously [10, 11]. Briefly, patients with nonmetastatic, resected, primary breast cancer, ≥ 4 involved axillary nodes, and no prior chemotherapy or radiotherapy were randomized to either ddCTX (four cycles of epirubicin plus paclitaxel followed by three cycles of cyclophosphamide, methotrexate, and 5‐fluorouracil [CMF], every 2 weeks) or stCTX (four cycles of epirubicin plus cyclophosphamide, followed by three cycles of CMF, every 3 weeks). Filgrastim was administered as primary prophylactic growth factor support in the ddCTX arm. Patients with HR‐positive disease received tamoxifen for 5 years. As the study was conducted in the pretrastuzumab era, HER2 status was not assessed, nor was anti‐HER2 therapy administered. Patients also received adjuvant radiotherapy following chemotherapy, according to national guidelines at that time point. The primary endpoint was disease‐free survival (DFS).

The clinical trial was conducted in accordance with the Declaration of Helsinki and had approval from independent ethics committees (IECs) at the study sites as well as written informed consent from patients [10, 11]. The principal site, Charité Mitte Hospital, Berlin, Germany, acted as the repository for tumor samples and the use of these for prospective exploratory analysis was approved by the IEC (Ethikkommission der Charité – Universitaetsmedizin Berlin, Berlin, Germany; Approval Number: 57/97).

### 2.2 Gene expression analysis

Messenger RNA (mRNA) was extracted from formalin‐fixed, paraffin‐embedded tumor specimens and analyzed using the Breast Cancer 360™ (BC360) panel version 2 on the multiplexed digital nCounter^®^ platform (NanoString Technologies, Inc., Seattle, WA, USA. https://nanostring.com/products/ncounter‐assays‐panels/oncology/breast‐cancer‐360/) [12]. Including 18 housekeeping genes, the BC360 panel comprises 758 genes relevant to breast cancer, and with established roles in tumor biology, the immune response, and the tumor microenvironment. Transcript counts were log_2_‐transformed and normalized to internal controls and housekeeping gene expression. For each sample, normalized data were used to determine correlation scores for the four Prosigna^®^ (NanoString Technologies, Inc.) intrinsic subtype signatures, assign intrinsic subtype, and calculate risk of recurrence (ROR) according to published methods [13]. Besides these parameters, the present analysis also included an additional 31 genes and signatures that are a preselected focus of the panel based on expected relevance for breast cancer biology [12].

### 2.3 Statistics

We used Cox proportional hazards regression to evaluate the prognostic and predictive relevance of intrinsic subtype and normalized gene/signature scores for DFS and overall survival (OS). For prognostic analyses, univariate models estimated the hazard ratio (HR) and associated 95% confidence interval (CI) per standard deviation increase in continuous score within each study arm. For predictive analyses, univariate models evaluated treatment effect (i.e., ddCTX versus stCTX) in subgroups using the median value as the cut‐point. Treatment‐by‐subgroup interaction tests assessed heterogeneity in treatment effect. Statistically significant univariate associations were evaluated in multivariate models incorporating clinicopathologic covariates (age [< 43 versus ≥ 43 years], pT stage [T1 versus T2 versus T3 versus T4], and number of involved nodes [4–9 versus > 9]) and/or intrinsic subtype (for overall population only). Survival curves were generated using the Kaplan–Meier estimator. Gene/signature scores were also compared between patients with or without either recurrence or mortality events during follow‐up (irrespective of treatment arm), using Student's *t*‐test (for normally distributed scores) or the Mann–Whitney *U*‐test (for non‐normally distributed scores). Scores with statistically significant univariate *P*‐values in these binary outcome analyses were further investigated by multiple logistic regression including clinicopathologic factors and/or intrinsic subtype. Univariate *P*‐values for gene/signature score associations were corrected for multiplicity within each set of analyses using the Benjamini–Hochberg false discovery rate (FDR) adjustment [14]. Alongside the main analyses in the overall population, prognostic and predictive effects were explored within intrinsic subtypes.

Statistical analyses were performed using spss Statistics Version 23.0 (IBM Corporation, Armonk, NY, USA). All analyses were exploratory and hypothesis generating.

## 3 Results

### 3.1 Patient population

**Fig. 1:**
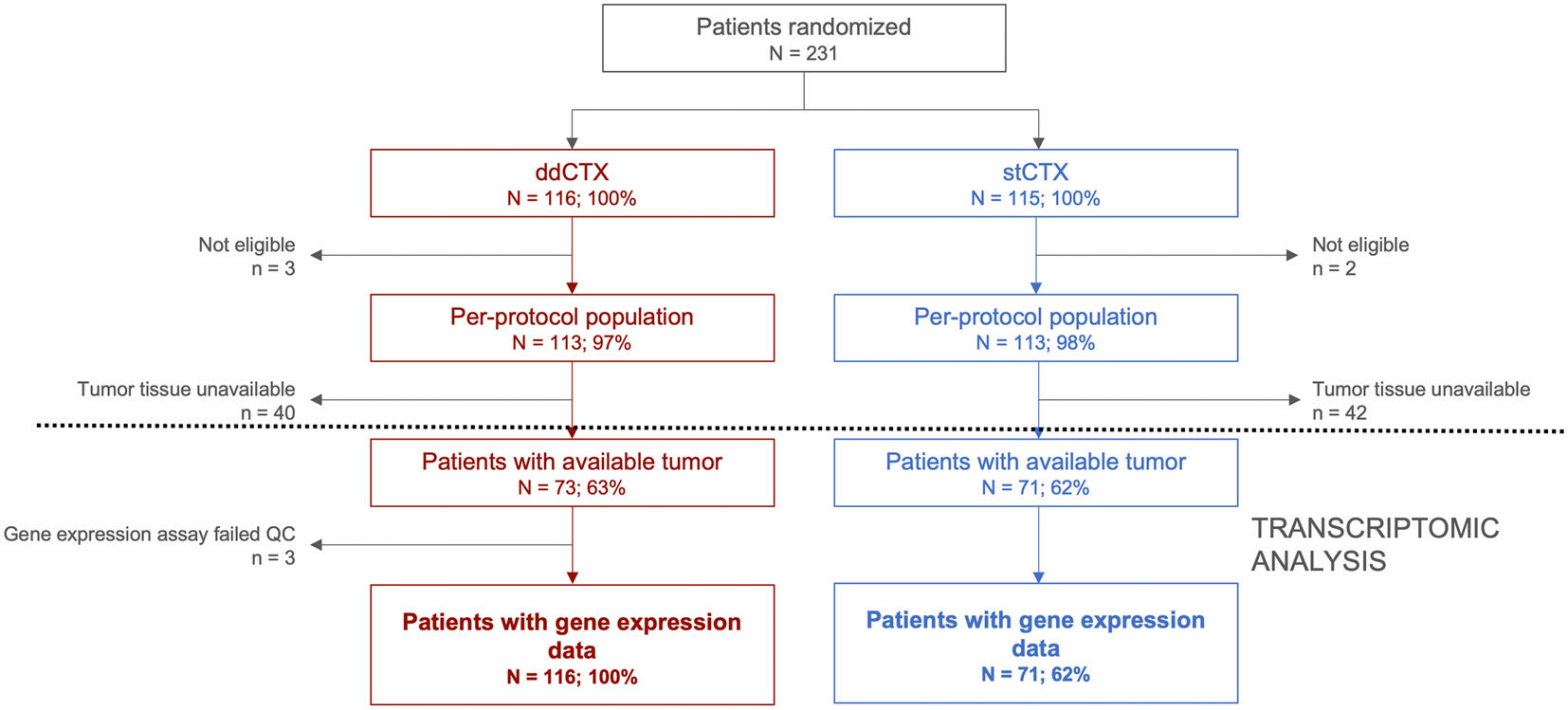
Flowchart depicting the study plan. The section above the dotted line shows patients as part of the reported clinical trial [11, 12] while the section under the dotted line constitutes our *post hoc* transcriptomic analysis. ddCTX, dose‐dense chemotherapy; stCTX, standard chemotherapy; QC, quality control.

Of 226 patients in the per‐protocol population (ddCTX, *N* = 113; stCTX, *N* = 113), 144 (63.7%) had tumor tissue available for transcriptomic analysis, which was successful for 141 patients (62.4%; ddCTX, *N* = 70; stCTX, *N* = 71; Fig. 1). The majority of these patients were postmenopausal (ddCTX, 68.6%; stCTX, 60.6%), had a T2 tumor (ddCTX, 48.6%; stCTX, 46.5%) and hormone receptor positive disease (ddCTX, 71.4%; stCTX, 81.7%), with 4 to 9 involved lymph nodes (ddCTX, 72.9%; stCTX, 76.1%). Baseline characteristics of patients with gene expression data resembled those of the per‐protocol population (Table 1).

**Table 1:** Patient characteristics from the per‐protocol population (*N* = 226) enrolled in the clinical trial [11, 12] and from sub‐cohort of patients (*N* = 141) whose tumors were analyzed using the BC360™ panel to obtain gene expression data. ddCTX, dose‐dense chemotherapy; stCTX, standard chemotherapy.

	Per‐protocol population			Patients with gene expression data		
	ddCTX (*N* = 113)	stCTX (*N* = 113)	*P* value^a^	ddCTX (*N* = 70)	stCTX (*N* = 71)	*P* value^a^
Median age, years (range)	55 (25–71)	55 (32–71)	0.875	55 (31–71)	56 (32–71)	0.800
Menopausal status, *N* (%)			0.892			0.320
Premenopausal	44 (38.9)	45 (39.8)		22 (31.4)	28 (39.4)	
Postmenopausal	69 (61.1)	68 (60.2)		48 (68.6)	43 (60.6)	
Laterality, *n* (%)			0.287			0.804
Left	63 (55.8)	55 (48.7)		36 (51.4)	38 (53.5)	
Right	50 (44.2)	58 (51.3)		34 (48.6)	33 (46.5)	
pT stage			0.799			0.965
1	33 (29.2)	28 (24.8)		17 (24.3)	18 (25.4)	
2	55 (48.7)	60 (53.1)		34 (48.6)	33 (46.5)	
3	20 (17.7)	19 (16.8)		12 (17.1)	14 (19.7)	
4	5 (4.4)	5 (4.4)		3 (4.3)	4 (5.6)	
Missing	0	1 (0.9)		4 (5.7)	2 (2.8)	
Median number of lymph nodes removed (range)	21 (8–38)	19 (8–92)	0.893	21 (9–38)	18 (8–92)	0.671
Number of involved lymph nodes, *N* (%)			0.438			0.890
4–9	83 (73.5)	88 (77.9)		51 (72.9)	54 (76.1)	
>9	30 (26.5)	25 (22.1)		15 (21.4)	15 (21.1)	
Missing	0	0		4 (5.7)	2 (2.8)	
Grade, *n* (%)			0.870			0.958
G1	7 (6.2)	6 (5.3)		4 (5.7)	4 (5.6)	
G2	54 (47.8)	53 (46.9)		34 (48.6)	34 (47.9)	
G3	48 (42.5)	52 (46.0)		28 (40.0)	31 (43.7)	
Missing	4 (3.5)	2 (1.8)		4 (5.7)	2 (2.8)	
Hormone receptor status, *N* (%)			0.251			0.150
Negative	27 (23.9)	20 (17.7)		20 (28.6)	13 (18.3)	
Positive	86 (76.1)	93 (82.3)		50 (71.4)	58 (81.7)	
Type of surgery, *N* (%)			0.684			0.857
Breast‐conserving surgery	44 (38.9)	47 (41.6)		22 (31.4)	22 (31.0)	
Mastectomy	69 (61.1)	66 (58.4)		44 (62.9)	47 (66.2)	
Missing	0	0		4 (5.7)	2 (2.8)	

a Categorical variable distributions were compared between arms using Fisher's exact test, while the means of continuous variables were compared using Student's *t*‐test.

Median follow‐up for patients with gene expression data, that is, patients whose tumor samples were available for gene expression analysis, was 14 years (95% confidence interval [CI]: 11–17). The significant improvement in DFS and the trend for longer OS with ddCTX versus stCTX previously observed in multivariate analyses of the per‐protocol population [14] were attenuated in patients with gene expression data (DFS – multivariate HR: 0.78, 95% CI: 0.48–1.27; *P* = 0.302; OS – multivariate HR: 0.80, 95% CI: 0.49–1.32; *P* = 0.375; Table S1). However, there were numeric trends favoring the ddCTX arm (adjusted 10‐year DFS: 52.7% with ddCTX, 44.8% with stCTX; adjusted 10‐year OS: 61.5% and 54.9%, respectively; Fig. S1).

### 3.2 Prognostic and predictive analyses of genes and signatures in overall population

Following univariate regression analyses, DFS in the ddCTX arm was found to have significant positive prognostic associations, that is, improved survival, with a number of genes and gene signatures. Following multivariate analysis, the associations remained statistically significant for: tumor inflammation signature (TIS; HR: 0.61, 95% CI: 0.41–0.91; *P* = 0.016), indoleamine 2,3‐dioxygenase‐1 (IDO1; HR: 0.78, 95% CI: 0.62–0.98; *P* = 0.032), programmed cell death 1 ligand‐1 (PD‐L1; HR: 0.62, 95% CI: 0.43–0.89; *P* = 0.010), programmed death‐ligand‐2 (PD‐L2; HR: 0.52, 95% CI: 0.35–0.77; *P* = 0.001), T‐cell immunoreceptor with immunoglobulin and immunoreceptor tyrosine‐based inhibition motif domain (TIGIT; HR: 0.73, 95% CI: 0.54–0.98; *P* = 0.038), CD8+ T‐cell signature (HR: 0.65, 95% CI: 0.45–0.93; *P* = 0.019), cytotoxic cell signature (HR: 0.62, 95% CI: 0.41–0.93; *P* = 0.020), and apoptosis signature (HR: 0.78, 95% CI: 0.61–0.99; *P* = 0.048; Table 2, Fig. S2A). In the stCTX arm, progesterone receptor (PGR) expression correlated with longer DFS (HR: 0.84, 95% CI: 0.74–0.96; *P* = 0.01) and p53 mutant‐like signature score correlated with a shorter DFS (HR: 1.33, 95% CI: 1.03–1.72; *P* = 0.026; Fig. S2A). Of all these associations, the PD‐L2 effect in the ddCTX arm retained statistical significance after FDR correction (*P* = 0.036). All univariate associations were significant after covariate adjustment, except for APM expression (Table 2).

**Table 2:** Multivariate analysis of prognostic associations for genes and signatures for DFS and OS in the overall population (*N* = 141). Multivariate analyses were performed only for variables attaining significance in univariate analyses. Statistically significant associations are shown in bold. APM, antigen processing machinery; CI, confidence interval; ddCTX, dose‐dense chemotherapy; DFS, disease‐free survival; ER, estrogen receptor; HER2, human epidermal growth factor receptor‐2; HR, hazard ratio; IDO1, indoleamine 2,3‐dioxygenase‐1; NA, not available; NS, not significant; OS, overall survival; PD‐L1, programmed death‐ligand‐1; PD‐L2, programmed death‐ligand‐2; PGR, progesterone receptor; ROR, risk of recurrence; stCTX, standard‐dosed chemotherapy; TIGIT, T‐cell immunoreceptor with immunoglobulin and immunoreceptor tyrosine‐based inhibition motif domains; TIS, tumor inflammation signature.

Arm, gene/signature	DFS		OS	
	HR (95% CI)^a^	*P* value	HR (95% CI)^a^	*P* value
ddCTX arm (*N* = 70)				
TIS	**0.61 (0.41–0.91)**	**0.016**	NA	NS
IDO1	**0.78 (0.62–0.98)**	**0.032**	NA	NS
PD‐L1	**0.62 (0.43–0.89)**	**0.010**	NA	NS
PD‐L2	**0.52 (0.35–0.77)**	**0.001**	NA	NS
TIGIT	**0.73 (0.54–0.98)**	**0.038**	NA	NS
CD8+ T‐cells	**0.65 (0.45–0.93)**	**0.019**	NA	NS
Cytotoxic cells	**0.62 (0.41–0.93)**	**0.020**	NA	NS
APM	0.71 (0.50–1.00)	0.051	NA	NS
Apoptosis	**0.78 (0.61–0.99)**	**0.048**	NA	NS
stCTX arm (*N* = 71)				
ROR	NA	NS	**1.03 (1.01–1.05)**	**0.017**
HER2‐enriched	NA	NS	**6.02 (1.86–19.5)**	**0.003**
Luminal A	NA	NS	**0.21 (0.09–0.53)**	**0.001**
PGR	**0.79 (0.68–0.91)**	**0.001**	**0.74 (0.63–0.87)**	**< 0.001**
Proliferation	NA	NS	**1.30 (1.04–1.64)**	**0.024**
p53 mutant‐like	**1.43 (1.05–1.96)**	**0.024**	**2.03 (1.40–2.94)**	**< 0.001**
ER signaling	NA	NS	**0.63 (0.44–0.91)**	**0.014**
Claudin‐low	NA	NS	**1.37 (1.00–1.86)**	**0.048**

a Adjusted for intrinsic subtype, age (< 43 versus ≥ 43 years), pT stage (T1 versus T2 versus T3 versus T4), and number of involved nodes (4–9 versus > 9).

In contrast, OS in the ddCTX arm was not found to have any statistically significant association with genes/signatures following univariate regression analyses (Fig. S2B). However, longer OS in the stCTX arm was found to correlate with PGR expression (HR: 0.82, 95% CI: 0.72–0.94; *P* = 0.005), estrogen receptor (ER) signaling (HR: 0.70, 95% CI: 0.51–0.98; *P* = 0.038), and luminal A signature (HR: 0.45, 95% CI: 0.23–0.86; *P* = 0.016). In contrast, shorter OS in the stCTX arm was found to correlate with ROR (HR: 1.03, 95% CI: 1.01–1.04; *P* = 0.01), HER2‐enriched (HER2‐E) signature (HR: 5.40, 95% CI: 1.93–15.10; *P* = 0.001), HER2 expression (HR: 1.23, 95% CI: 1.04–1.46; *P* = 0.016), proliferation score (HR: 1.21, 95% CI: 1.02–1.45; *P* = 0.033), p53 mutant‐like (HR: 1.48, 95% CI: 1.15–1.92; *P* = 0.003), and claudin‐low signatures (HR: 1.38, 95% CI: 1.02–1.88; *P* = 0.038). Of these, the HER2‐enriched signature effect was significant after FDR adjustment (*P* = 0.036). Associations remained statistically significant following multivariate analyses (Table 2).

In an analysis of recurrence and mortality as binary outcomes, we found recurrence to be associated with significantly lower PD‐L1/2 expression and CD8+ T‐cell and cytotoxic cell abundance (Table 3). In addition, mortality was associated with significantly lower CD8+ T‐cell score and inflammatory chemokine expression. These differences were not significant after FDR adjustment.

**Table 3:** Analysis of recurrence and mortality as binary outcomes on the overall population and according to intrinsic subtype. Statistically significant associations are shown in bold. Only genes/signatures with significant unadjusted *P* values for at least one outcome are shown. APM, antigen processing machinery; BRCA, Breast Cancer gene; FDR, false‐discovery rate; HER2, human epidermal growth factor receptor‐2; IDO1, indoleamine 2,3‐dioxygenase‐1; PD‐1, programmed death‐1; PD‐L1, programmed death‐ligand‐1; PD‐L2, programmed death‐ligand‐2; PGR, progesterone receptor; SD, standard deviation; TIGIT, T‐cell immunoreceptor with immunoglobulin and immunoreceptor tyrosine‐based inhibition motif domains; TIS, tumor inflammation signature.

	Recurrence			Mortality		
	Mean (SD) score		*P*‐value	Mean (SD) score		*P*‐value
	Patients with event	Patients without event		Patients with event	Patients without event	
Overall (*N* = 141)						
PD‐L1	−3.38 (0.92)	−2.90 (1.05)	**0.005**	−3.30 (0.94)	−3.00 (1.06)	0.074
PD‐L2	−3.67 (0.92)	−3.32 (0.88)	**0.022**	−3.56 (0.86)	−3.44 (0.96)	0.435
CD8+ T‐cells	−2.54 (0.97)	−2.18 (1.04)	**0.037**	−2.53 (0.96)	−2.20 (1.05)	**0.048**
Cytotoxic cells	−3.62 (0.94)	−3.27 (1.06)	**0.039**	−3.58 (0.95)	−3.33 (1.05)	0.131
Inflammatory chemokines	4.31 (1.29)	4.53 (1.09)	0.280	4.21 (1.22)	4.62 (1.14)	**0.037**
Luminal A (*N* = 49)						
Basal	−0.42 (0.29)	−0.57 (0.20)	**0.031** ^**a**^	−0.42 (0.29)	−0.57 (0.30)	0.132
p53	4.28 (0.84)	3.74 (0.77)	**0.028**	4.28 (0.84)	3.74 (0.79)	**0.028**
Differentiation	5.17 (0.73)	5.58 (0.90)	**0.006**	5.17 (0.73)	5.58 (0.90)	0.056
BRCAness	4.75 (0.76)	4.43 (0.57)	**0.013**	4.75 (0.76)	4.43 (0.77)	0.099
Luminal B (*N* = 39)						
PGR	−1.59 (2.47)	−0.59 (1.58)	0.242	−1.90 (2.37)	−0.28 (1.61)	**0.015** ^**a**^
Inflammatory chemokines	3.78 (0.90)	4.50 (0.65)	**0.012**	3.88 (0.85)	4.26 (0.92)	0.196
HER2‐enriched (*N* = 27)						
Inflammatory chemokines	3.67 (1.39)	4.50 (1.28)	0.120	3.52 (1.39)	4.69 (1.10)	**0.025**
Basal‐like (*N* = 26)						
TIS	6.99 (0.80)	8.39 (0.62)	**<0.001** ^**a**^ ^,^ ^**b**^	7.18 (0.81)	8.32 (0.84)	**0.002** ^**a**^ ^,^ ^**b**^
IDO1	−1.99 (1.12)	0.12 (1.63)	**0.001** ^**b**^	−1.96 (1.19)	0.25 (1.55)	**<0.001** ^**a**^ ^,^ ^**b**^
PD‐L1	−3.24 (0.86)	−1.86 (0.70)	**<0.001** ^**a**^ ^,^ ^**b**^	−3.04 (0.98)	−1.95 (0.81)	**0.005** ^**a**^ ^,^ ^**b**^
PD‐L2	−3.48 (0.90)	−2.66 (0.96)	**0.036**	−3.41 (0.87)	−2.67 (1.03)	0.060
TIGIT	−3.49 (0.93)	−2.35 (0.90)	**0.004** ^**b**^	−3.32 (0.96)	−2.44 (1.02)	**0.033**
CD8+ T‐cells	−2.38 (0.50)	−1.45 (0.81)	**0.002** ^**b**^	−2.34 (0.55)	−1.41 (0.79)	**0.002** ^**a**^ ^,^ ^**b**^
Cytotoxic cells	−3.59 (0.85)	−2.39 (0.69)	**0.001** ^**a**^ ^,^ ^**b**^	−3.38 (0.77)	−2.51 (0.98)	**0.018**
APM	5.66 (1.15)	6.70 (0.89)	**0.016**	5.79 (1.15)	6.64 (0.97)	0.055
Cytotoxicity	5.09 (1.55)	6.50 (1.40)	**0.022**	5.28 (1.59)	6.43 (1.46)	0.067
PD‐1	−4.70 (1.09)	−3.20 (0.85)	**0.001** ^**a**^ ^,^ ^**b**^	−4.50 (1.11)	−3.29 (1.03)	**0.009** ^**a**^

a Multiple logistic regression adjusted for intrinsic subtype (overall population only), age (< 43 versus ≥ 43 years), pT stage (T1 versus T2 versus T3 versus T4), and number of involved nodes (4–9 versus > 9).

b *P*‐value < 0.05 following FDR correction.

**Fig. 2:**
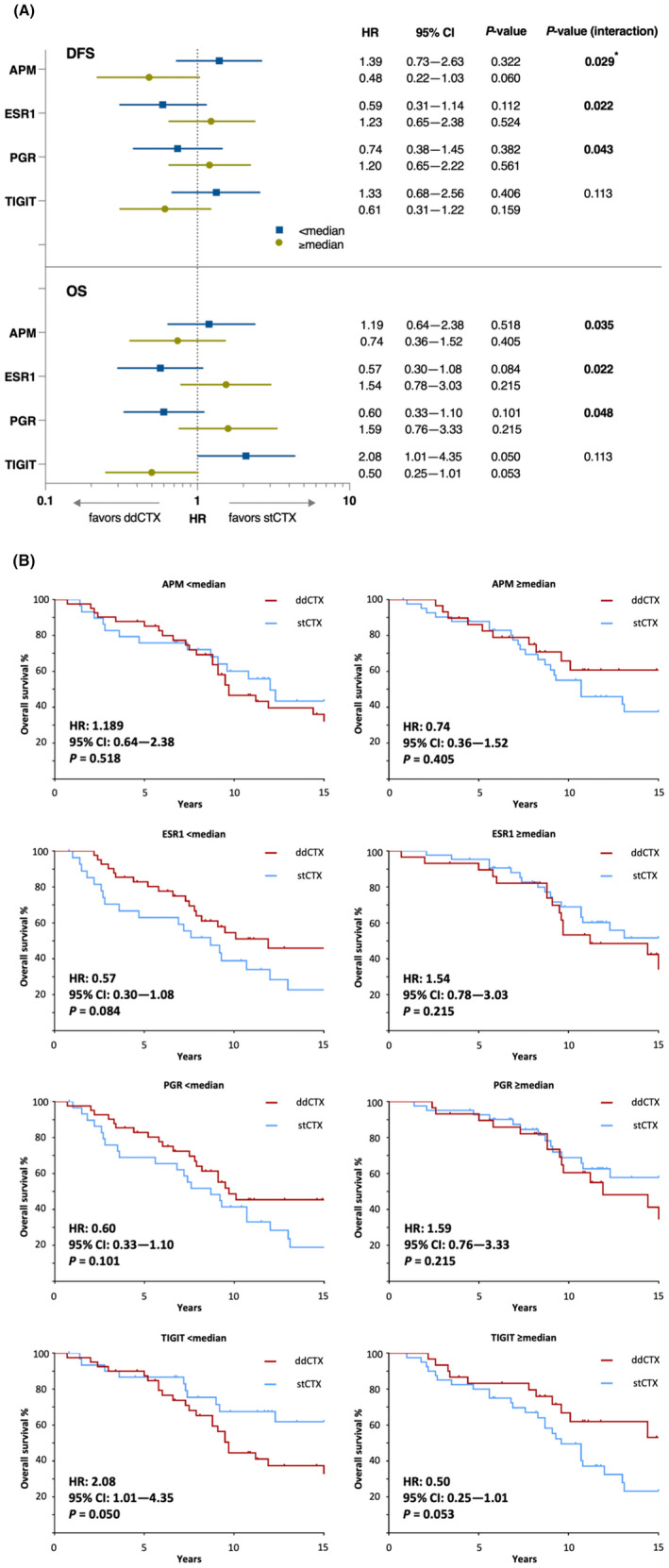
Predictive analysis for interaction between genes/signature and treatment intensity identified APM, ESR1 and PGR as genes the expression of which (≥ median or < median) is associated with effectiveness of treatment. (A) ddCTX was associated with a better treatment effect in tumors that had low (< median) expression of ESR1 or PGR and high (≥ median) expression of APM. (B) These trends appear to be reflected in Kaplan–Meier analysis of OS although the differences between both treatments were not statistically significant. Although difference in OS was the strongest for TIGIT expression, the genes/signature‐by‐treatment effect was not statistically significant. Multivariate analyses were performed only for variables with significant unadjusted *P* values either for treatment‐by‐gene/signature interaction or for treatment effect within a gene/signature subgroup. Kaplan–Meier survival curves were analyzed using Cox regression analysis and Wald test. Statistically significant associations are shown in bold. Asterisk (*) indicates statistical significance after multivariate analysis. APM, antigen processing machinery; CI, confidence interval; ddCTX, dose‐dense chemotherapy; PFS, progression‐free survival; ESR1, estrogen receptor‐1; HR, hazard ratio; OS, overall survival; PGR, progesterone receptor; stCTX, standard‐dosed chemotherapy; TIGIT, T‐cell immunoreceptor with immunoglobulin and immunoreceptor tyrosine‐based inhibition motif domains.

Predictive effects, following assessment in treatment‐by‐gene/signature interaction in bivariate models, were statistically significant in analyses of both DFS and OS for three gene expression variables: estrogen receptor 1 (ESR1), PGR, and APM (Fig. 2A; Fig. S3); treatment effect HRs indicated better treatment effect of ddCTX versus stCTX in patients whose tumors had low (< median) ESR1 or PGR and high (≥ median) APM expression. Despite a substantial difference in treatment effect in between treatment and TIGIT expression, indicated by the HR in Kaplan–Meier survival analysis (Fig. 2B), the interaction between TIGIT expression and treatment was not statistically significant after bivariate analysis. Following adjustment for covariates, treatment‐by‐gene/signature interaction was significant only for APM (*P* = 0.024).

### 3.3 Prognostic and predictive analysis of genes and signatures according to intrinsic subtype

The majority of tumors were of intrinsic subtype luminal A (*n* = 49, 34.8%), followed by luminal B (*n* = 39, 27.7%), HER2‐E (*n* = 27, 19.1%), and basal‐like (*n* = 26, 18.4%).

**Fig. 3:**
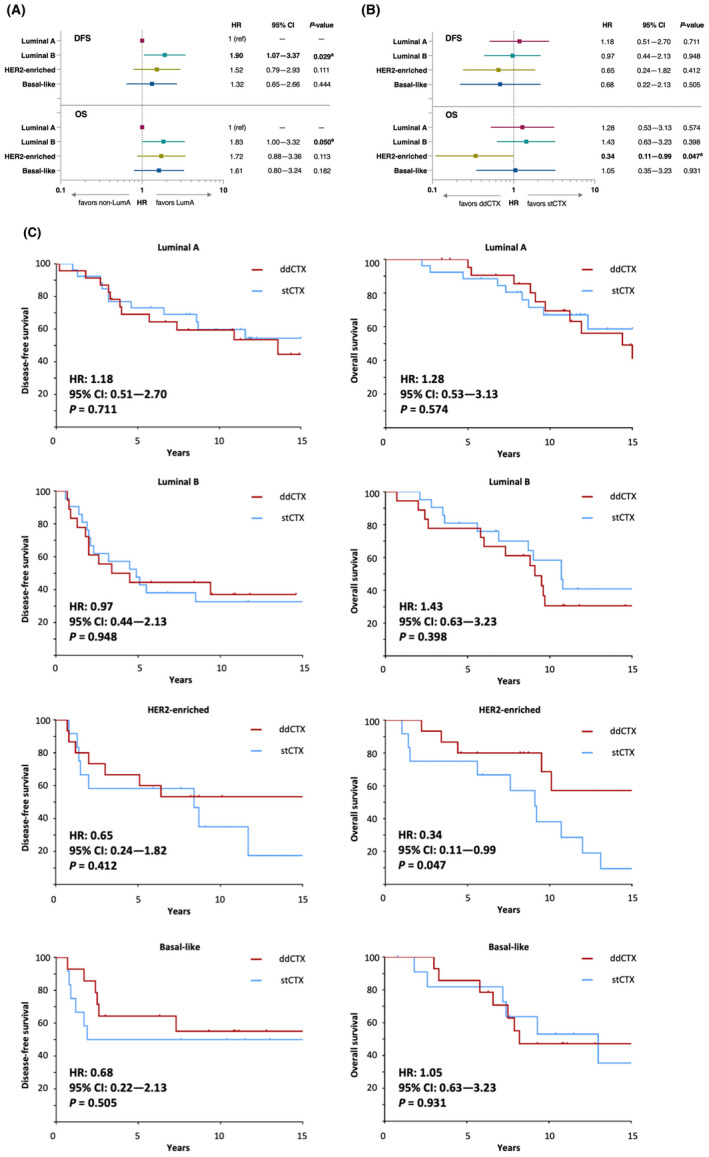
Intrinsic subtype of tumors is found to have prognostic and predictive association with survival outcomes. (A) In comparison with patients with Luminal A tumors, patients with other intrinsic subtypes had poorer survival outcomes which were statistically significant in patients with Luminal B tumors. (B) ddCTX showed a better treatment effect in comparison with stCTX in patients with HER2‐E tumors, (C) as seen also in the Kaplan–Meier survival analysis of the OS in these patients. Multivariate analyses were performed only for variables with significant unadjusted *P* values either for treatment‐by‐gene/signature interaction or for treatment effect within a gene/signature subgroup. Kaplan–Meier survival curves were analyzed using Cox regression analysis and Wald test. Statistically significant associations in (A) and (B) are shown in bold. ^a^ *P*‐value < 0.05 following multivariate analyses adjusting for age (< 43 versus ≥ 43 years), pT stage (T1 versus T2 versus T3 versus T4), number of involved nodes (4–9 versus > 9) and hormone receptor status (positive versus negative). CI, confidence interval; ddCTX, dose‐dense chemotherapy; DFS, disease‐free survival; HER2, human epidermal growth factor receptor‐2; HR, hazard ratio; OS, overall survival; stCTX, standard dose chemotherapy.

Although all other intrinsic subtypes in comparison with luminal A tumors had poorer DFS and OS, a statistically significant reduction in DFS (*P* = 0.029) and OS (*P* = 0.05) both before and after adjustment for clinical covariates was only evident in patients with luminal B tumors (Fig. 3A).

Following predictive analyses, ddCTX was found to improve DFS and OS numerically in comparison with stCTX in patients with HER2‐E tumors, although statistical significance after adjustment for covariates was achieved only for OS (Fig. 3B). This treatment effect is better demonstrated through a Kaplan–Meier plot; OS was significantly longer with ddCTX in comparison with stCTX in the HER2‐E subtype (Fig. 3C). Treatment‐by‐subtype interactions were not statistically significant for either DFS or OS.

Following univariate analysis of gene/signature expression data in subgroups comprising patients with tumors of each intrinsic subtype, several genes/signatures showed a significant association at both prognostic and predictive levels with DFS and OS (Figs S4–S11). However, with a few exceptions, a number of these associations failed to hold statistical significance following either FDR correction or multivariate analysis.

In patients with luminal A tumors, low expression of gene signature representing stroma was associated with improved survival outcomes for both DFS and OS in the stCTX arm (DFS – HR: 2.13, 95% CI: 0.54–8.33; *P* (interaction) < 0.001; OS – HR: 2.33, 95% CI: 0.58–9.09; *P* < 0.001). This association was found to be statistically significant following FDR (*P*_DFS_ = 0.004, *P*_OS_ = 0.007; Fig. S5).

Similarly, in patients with HER2‐E tumors, high expression of androgen receptor (AR) and mast cell signature was associated with poorer OS in the ddCTX arm. On the contrary, for patients in the stCTX arm, tumoral expression of signatures coding CD8+ T cells and cytotoxic cells correlated with improved survival outcomes (longer DFS and OS with CD8+ signature and longer DFS with cytotoxic cell signature). Following multivariate analysis adjusted for clinicopathologic covariates, these associations were found to be statistically significant (Table S2). Although low expression of inflammatory chemokines was found to be associated with improved OS in patients who had received ddCTX (Table 3, Fig. S9), this association could not be replicated after FDR correction. However, treatment‐by‐gene/signature interaction of inflammatory chemokines with both DFS and OS was found to be statistically significant following multivariate analysis (Table S3).

Lastly, in patients with basal‐like tumors, high expression of TIS and PD‐L1 expression was found to be associated with longer DFS and OS in the ddCTX arm. Moreover, expression of IDO1, TIGIT, CD8+ T‐cells, cytotoxic cells, and PD‐1 were associated with improvement in OS, and cytotoxicity cell signature with improvement in DFS in patients treated with ddCTX. Following FDR adjustment, associations in basal‐like tumors remained significant for TIS, IDO1, PD‐L1 and CD8+ T‐cells (recurrence and mortality) and TIGIT, cytotoxic cells and PD‐1 (recurrence only). Associations that showed significant FDR‐corrected univariate *P* values were also significant after adjustment for covariates (Table 3). Interestingly, in this subgroup with basal‐like tumors, high expression of ESR1 was associated with improved DFS in the ddCTX arm; an association that remained significant after multivariate analysis (*P* = 0.045; Table S4).

## 4 Discussion

There is a marked unmet need for biomarkers to assist in the formulation of rational treatment guidance and de‐escalation strategies in patients with EBC and ≥ 4 positive nodes, most of whom currently receive chemotherapy. Multigene expression profiles have demonstrated prognostic and predictive relevance in patients with 0–3 positive nodes [4, 5, 6, 7, 8], but data in more advanced disease are limited. To our knowledge, we report the first assessment of a large panel of transcriptomic biomarkers in tumors obtained from a randomized phase 3 adjuvant trial of patients with EBC and ≥ 4 involved nodes, for whom an extended median follow‐up of 14 years provided comprehensive data on long‐term outcome. We identified several genes/signatures with prognostic significance, as well as potential predictive factors for taxane‐containing ddCTX, in an EBC patient cohort not treated with HER2‐directed therapy, platinum agents or immunotherapy.

Previous phase 3 trials in mostly early‐stage disease have shown an association between nonluminal intrinsic subtypes and beneficial outcomes from taxane‐ and anthracycline‐containing ddCTX [15, 16], but not from addition of weekly paclitaxel to stCTX [17]. The greater sensitivity of the more proliferative subtypes to ddCTX could be predicted based on the Norton–Simon hypothesis, which states that the rate of tumor regression following chemotherapy is directly proportional to the rate of tumor growth [18]. Certain large‐scale meta‐analyses [1, 2] have shown a lack of association between standard clinical breast cancer subtypes and efficacy of add‐on taxane or increased dose density. In contrast, other studies have demonstrated HER2‐positivity and ER‐negativity to be correlated with improved outcomes for taxane‐based regimen and ddCTX, respectively [19, 20, 21, 22]. Our analysis adds to the growing evidence from phase 3 trials that the intrinsic subtype could have an independent prognostic relevance [23]. In addition, our results further suggest a greater efficacy of taxane‐containing ddCTX in comparison with nontaxane‐containing stCTX in nonluminal subtypes, observed as a statistically significant OS benefit especially in HER2‐E tumors. While these results support the use of such regimens for HER2‐E tumors, especially when access to anti‐HER2 therapies is limited; these effects would also require validation through prospective phase 3 trials given recent conflicting findings on the effectiveness of ddCTX combined with anti‐HER2 therapy for HER2‐positive EBC [24, 25].

Innate antitumor immune responses, reflected by lymphocytic infiltration of the tumor mass, prior to therapeutic intervention, has been found to positively influence underlying prognosis in HER2‐positive and triple‐negative EBC [26, 27, 28, 29]. A crucial consequence of immune activation is the release of interferon‐gamma into the tumor microenvironment, which subsequently potentiates immune responses (e.g., by enhancing APM expression) and triggers adaptive immunosuppressive mechanisms (e.g., by inducing expression of checkpoint proteins such as PD‐L1/2, TIGIT and IDO1) [30]. Paradoxically, these latter effects are also known to contribute to immune escape, which is correlated with both the development and progression of nodal and distant metastases [26, 29, 31]. In our analysis, we discovered that in patients with advanced nodal stage, several transcriptomic measures of immunity—including TIS, CD8+ T‐cell and cytotoxic cell abundance, and expression of PD‐L1/2, TIGIT and IDO1—were independently and positively associated with improved DFS in the ddCTX arm. Of these associations, PD‐L2 had the strongest effect, which remained significant after multiplicity correction. Whereas a favorable effect of PD‐L1 mRNA expression on survival in breast cancer has been established in a large meta‐analysis [32], the prognostic relevance of PD‐L2 is yet to be understood completely. Prior retrospective analyses have failed to ascertain the impact of PD‐L2 gene expression on survival outcomes [33, 34] although an association for PD‐L2 mRNA was shown before multiplicity correction in triple‐negative EBC treated with capecitabine‐containing adjuvant therapy [35]. A recent analysis of unselected breast cancers in the UALCAN database also reported favorable prognostic effects of TIGIT and IDO1 expression [36]. PD‐L1/2, TIGIT, and IDO1—all included within the 18‐gene TIS, which was developed as a pan‐cancer predictor of response to pembrolizumab [30]—were also found to independently predict improvement in DFS in the ddCTX arm of our analysis. Interestingly, in prior studies, TIS was not found to be prognostic for OS in unselected breast cancers [37] or triple negative metastatic breast cancer [38] but was associated with longer recurrence‐free survival in triple negative patients receiving capecitabine containing adjuvant treatment [35].

The immune response prior to commencement of chemotherapy is also thought to make important contributions to the clinical efficacy of conventional chemotherapeutic agents [39], but the role of specific immune markers in sensitivity to particular drugs and regimens remains unclear. In translational analyses of previous phase 3 adjuvant trials, the presence of tumor‐infiltrating lymphocytes (TILs) was found to be predictive of greater efficacy of anthracycline‐only versus anthracycline‐ and docetaxel‐containing chemotherapy in HER2‐positive disease [40]; intensified, dose‐dense epirubicin, paclitaxel, and cyclophosphamide in node‐positive EBC [41]; and stCTX with docetaxel, doxorubicin, and cyclophosphamide in triple‐negative disease [42]. TILs and/or immune gene expression (including PD‐L1) are also implicated in sensitivity to carboplatin‐containing neoadjuvant regimens for HER2‐positive or triple‐negative tumors [43, 44]. The arm‐specificity of many of the associations observed in our analysis is suggestive of predictive effects, and we formally demonstrated a treatment‐by‐gene/signature interaction for APM for DFS in multivariate analysis, along with a possible trend for TIGIT. Interestingly, when measured using the same transcriptomic panel as we did (BC360), PD‐L2 expression and the abundance of cytotoxic and mast cells were shown to predict efficacy of adding capecitabine to anthracycline‐ and taxane‐containing stCTX in a recent analysis of triple‐negative patients in the FinXX study [35]. Taking into consideration these observations, one could speculate that increased immunogenic cell death resulting from greater dose density and/or addition of a taxane or capecitabine may have a greater effect in patients with stronger pretreatment immune responses, that is, immunologically ‘hot’ tumors [39].

The need for novel biomarkers is perhaps most acute in patients with HER2‐positive or triple‐negative disease, whose treatment burden may include HER2‐directed therapies, platinum agents and/or immunotherapies. We identified several prognostic associations for non‐luminal intrinsic subtypes in patients not exposed to these drug classes. Consistent with the greater immunogenicity of basal‐like tumors [29], the positive impact of immune genes/signatures was particularly pronounced in the basal‐like subgroup. Interestingly, there was also a strong, independent, positive DFS effect for ESR1 expression in ddCTX‐treated basal‐like tumors. ER‐positivity in tumors with basal‐like transcriptional subtype has been associated with the expression of dominant‐negative ER variants [45], and improved outcome relative to ER‐negative basal‐like tumors [46]. In HER2‐enriched tumors, AR expression and mast cell abundance were negatively correlated with survival, for which there is some precedence in the literature [47, 48]. With the development of immunotherapies for EBC, an understanding of how immunologic factors affect response to chemotherapeutic partner agents will be increasingly important to help optimize use of novel drugs.

Our analysis, although detailed, is not without limitations, of which a major one is the *post hoc* and exploratory nature of the analysis. In addition, our analysis was conducted on primary tumor samples collected in the pretrastuzumab era and without assessment of HER2 expression thus failing to account for the change in treatment landscape since the introduction of trastuzumab for treatment of HER2‐positive EBC. Although transcriptomic analysis enabled evaluation of HER2‐E and basal‐like tumors, these classifications are only partially concordant with HER2‐positive and triple‐negative clinical subtypes [23]. Our attempt at trying to identify predictive and prognostic biomarkers could have potentially masked correlations or led to chance finding on account of smaller sample sizes of the subcohorts. This assumption is supported by wide confidence intervals for several genes and gene signatures, and it is quite likely that such issues could not be resolved despite conducting multivariate analysis and FDR corrections. Administration of paclitaxel in only the ddCTX arm precluded differentiation between predictive effects relating to taxane addition versus increased dose density, while CMF is no longer considered a standard of care. Thus, our findings will need to be validated in other independent cohorts in order to identify appropriate biomarkers that will guide the choosing of optimal adjuvant chemotherapy. We expect such an approach to be promising as evidenced from a recently reported analysis of the phase 3 CALGB9741 (Alliance) trial. Here, the investigators discovered that expression of SET2,3, a biomarker of endocrine sensitivity, could have prognostic and predictive potential for the use of ddCTX in pre‐and postmenopausal women with ER‐positive cancer [49].

## 5 Conclusion

In conclusion, our analysis shows that gene expression assays have the potential to provide a valuable insight into the impact of gene expression in tumors and their microenvironment on long‐term outcome in patients with EBC and advanced nodal stage who receive adjuvant chemotherapy. If prospectively validated in future studies, the observed prognostic associations could form a basis for selection of patients for de‐escalated treatment strategies and thereby reduce the treatment burden in this otherwise heavily treated population. Furthermore, the predictive potential of immune marker expression in non‐luminal intrinsic subtypes receiving taxane‐containing ddCTX warrants further investigation.

## Conflict of interest

MR reports honoraria (self) and advisory/consultancy fees from Roche, Novartis, MSD, AstraZeneca, Seagen, Somatex, and Lilly; and travel expenses from Novartis, Celgene, and Pfizer. SB, DG, AT has declared no conflict of interest. OG reports minority non‐profit ownership of WSG Study Group; honoraria from Genomic Health, Roche, Celgene, Pfizer, Novartis, NanoString Technologies, and AstraZeneca; consulting/advisory board roles at Amgen, Roche, Daiichi Sankyo, Genomic Health, Merck Sharp & Dohme, Lilly, Novartis, Astra Zeneca, and Eisai; and travel/accommodation/expenses from Roche, Celgene, Daiichi Sankyo. BA reports advisory fees from Roche, Amgen, Tesaro; lecture honoraria from Roche, Tesaro, Celgene, Clovis, Astra Zeneca; and travel/accommodation expenses from Roche, Tesaro, and PharmaMar. PS reports advisory/consultancy fees from AstraZeneca, Bayer, Boehringer Ingelheim, Merck, Novartis, Pfizer, Puma, and Roche; grant/funding to the institution from Astellas, AstraZeneca, Genentech, Novartis, Oncogenex and Roche; and a spouse who is an employee of Roche. JC reports advisory role/consultancy for Roche, Celgene, Cellestia, AstraZeneca, Biothera Pharmaceutical, Merus, Seattle Genetics, Daiichi Sankyo, Erytech, Athenex, Polyphor, Lilly, Servier, Merck Sharp&Dohme, GSK, Leuko, Bioasis, Clovis Oncology, and Boehringer Ingelheim; honoraria from Roche, Novartis, Celgene, Eisai, Pfizer, Samsung Bioepis, Lilly, Merck Sharp & Dohme, and Daiichi Sankyo; research funding to the institution from Roche, Ariad Pharmaceuticals, AstraZeneca, Baxalta GMBH/Servier Affaires, Bayer Healthcare, Eisai, F. Hoffman‐La Roche, Guardant Health, Merck Sharp & Dohme, Pfizer, Piqur Therapeutics, Puma C, and Queen Mary University of London; stock, patents and intellectual property from MedSIR; and travel, accommodation, expenses from Roche, Novartis, Eisai, Pfizer, and Daiichi Sankyo. J‐UB reports advisory and presentation fees from Amgen, AstraZeneca, MSD, Novartis, Pfizer, Roche, SonoScape, Sysmex. MHD reports personal fees from Boehringer Ingelheim, Merck, Novartis, EUSA Pharma, AbbVie, Janssen, Biogen, Menarini and Norgine (outside the submitted work). SS reports professional fees from Sanofi, Abbvie, Bayer, Cantargia, Celgene, Ferring, Merus, Nestle, Servier, Tiburio, and Zentiva on unrelated freelance projects. CD‐G reports honoraria from AstraZeneca. OC reports travel expenses from Pfizer. HH reports travel expenses from Pfizer and Teva. SK reports consulting fees from F. Hoffmann‐La Roche Ltd, Genomic Health, Novartis, Amgen, Celgene, Daiichi Sankyo, AstraZeneca, Somatex, MSD, Pfizer, Puma Biotechnology, PFM Medical, Lilly and SonoScape; leadership roles with Westdeutsche Studiengruppe and Arbeitsgemeinschaft Gynäkologische Onkologie; research grant/funding from Somatex and Roche; travel expenses from F. Hoffmann‐La Roche Ltd, Daiichi‐Sankyo and SonoScape; and partial non‐profit ownership of Westdeutsche Studiengruppe.

## Author contributions

MR, SM, and SK were responsible for conceptualization, investigation, methodology, and overview of the study. SS and MHD were responsible for data curation, visualization, and writing of the manuscript. AT was responsible for statistical analysis of study data. All other co‐authors provided key inputs on study conduct, data analysis, and manuscript drafting.

### Peer review

The peer review history for this article is available at https://www.webofscience.com/api/gateway/wos/peer‐review/10.1002/1878‐0261.13435.

## Supporting information


**Table S1.** Univariate and multivariate analyses of DFS and OS in patients with gene expression data (*N* = 141).
**Table S2.** Multivariate analysis of prognostic associations for genes/signatures for DFS and OS in patients with HER2‐enriched tumors (*N* = 27).
**Table S3.** Multivariate analysis of predictive associations for genes/signatures for DFS and OS in patients with HER2‐enriched tumors (*N* = 27).
**Table S4.** Multivariate analysis of predictive associations for genes/signatures for DFS and OS in patients with basal‐like tumors (*N* = 26).
**Fig. S1.** Adjusted survival curves for (A) DFS and (B) OS in patients with gene expression data (*N* = 141).
**Fig. S2.** Prognostic analysis of genes and signatures according to treatment arm in overall population (*N* = 141) for (A) DFS and (B) OS.
**Fig. S3.** Predictive analysis of genes and signatures in the overall population (*N* = 141) for (A) DFS and (B) OS.
**Fig. S4.** Prognostic analysis of genes and signatures in patients with luminal A tumors (*N* = 49) for (A) DFS and (B) OS.
**Fig. S5.** Predictive analysis of genes and signatures in patients with luminal A tumors (*N* = 49) for (A) DFS and (B) OS.
**Fig. S6.** Prognostic analysis of genes and signatures in patients with luminal B tumors (*N* = 39) for (A) DFS and (B) OS.
**Fig. S7.** Predictive analysis of genes and signatures in patients with luminal B tumors (*N* = 39) for (A) DFS and (B) OS.
**Fig. S8.** Prognostic analysis of genes and signatures in patients with HER2‐enriched tumors (*N* = 27) for (A) DFS and (B) OS.
**Fig. S9.** Predictive analysis of genes and signatures in patients with HER2‐enriched tumors (*N* = 27) for (A) DFS and (B) OS.
**Fig. S10.** Prognostic analysis of genes and signatures according to treatment arm in patients with basal‐like tumors (*N* = 26) for (A) DFS and (B) OS.
**Fig. S11.** Predictive analysis of genes and signatures according to treatment arm in patients with basal‐like tumors (*N* = 26) for (A) DFS and (B) OS.

## Data Availability

Data supporting the findings of this study are available in Tables 1, 2, 3 and Figs 1, 2, 3 as well as in the Supporting Information section.
