# Supplementary material for: Prognostic and predictive impact of gene expression in node‐positive early breast cancer patients receiving dose‐dense versus standard‐dose adjuvant chemotherapy
Source: Mol Oncol. 2023 Apr 24;17(6):1060–75. doi: 10.1002/1878-0261.13435 (PMC10257423; doi:10.1002/1878-0261.13435)
Supplement: Supplementary file 1 — Table S1. Univariate and multivariate analyses of DFS and OS in patients with gene expression data (N = 141). Table S2. Multivariate analysis of prognostic associations for genes/signatures for DFS and OS in patients with HER2‐enriched tumors (N = 27). Table S3. Multivariate analysis of predictive associations for genes/signatures for DFS and OS in patients with HER2‐enriched tumors (N = 27). Table S4. Multivariate analysis of predictive associations for genes/signatures for DFS and OS in patients with basal‐like tumors (N = 26). Fig. S1. Adjusted survival curves for (A) DFS and (B) OS in patients with gene expression data (N = 141). Fig. S2. Prognostic analysis of genes and signatures according to treatment arm in overall population (N = 141) for (A) DFS and (B) OS. Fig. S3. Predictive analysis of genes and signatures in the overall population (N = 141) for (A) DFS and (B) OS. Fig. S4. Prognostic analysis of genes and signatures in patients with luminal A tumors (N = 49) for (A) DFS and (B) OS. Fig. S5. Predictive analysis of genes and signatures in patients with luminal A tumors (N = 49) for (A) DFS and (B) OS. Fig. S6. Prognostic analysis of genes and signatures in patients with luminal B tumors (N = 39) for (A) DFS and (B) OS. Fig. S7. Predictive analysis of genes and signatures in patients with luminal B tumors (N = 39) for (A) DFS and (B) OS. Fig. S8. Prognostic analysis of genes and signatures in patients with HER2‐enriched tumors (N = 27) for (A) DFS and (B) OS. Fig. S9. Predictive analysis of genes and signatures in patients with HER2‐enriched tumors (N = 27) for (A) DFS and (B) OS. Fig. S10. Prognostic analysis of genes and signatures according to treatment arm in patients with basal‐like tumors (N = 26) for (A) DFS and (B) OS. Fig. S11. Predictive analysis of genes and signatures according to treatment arm in patients with basal‐like tumors (N = 26) for (A) DFS and (B) OS. [file MOL2-17-1060-s001.docx]

[**Supplementary Table 1. Univariate and multivariate analyses of DFS and OS in patients with gene expression data (*N* = 141)** 2](#_Toc118821214)

[**Supplementary Table 2. Multivariate analysis of prognostic associations for genes/signatures for DFS and OS in patients with HER2-enriched tumors (*N* = 27)** 3](#_Toc118821215)

[**Supplementary Table 3. Multivariate analysis of predictive associations for genes/signatures for DFS and OS in patients with HER2-enriched tumors (*N* = 27)** 4](#_Toc118821216)

[**Supplementary Table 4. Multivariate analysis of prognostic associations for genes/signatures for DFS and OS in patients with basal-like tumors (*N* = 26)** 5](#_Toc118821217)

[**Supplementary Figure 1. Adjusted survival curves for (A) DFS and (B) OS in patients with gene expression data (*N* = 141)** 6](#_Toc118824594)

[**Supplementary Figure 2. Prognostic analysis of genes and signatures according to treatment arm in overall population (*N* = 141) for (A) DFS and (B) OS.** 7](#_Toc118824595)

[**Supplementary Figure 3. Predictive analysis of genes and signatures in the overall population (*N* = 141) for (A) DFS and (B) OS.** 9](#_Toc118824596)

[**Supplementary Figure 4. Prognostic analysis of genes and signatures in patients with luminal A tumors (*N* = 49) for (A) DFS and (B) OS.** 11](#_Toc118824597)

[**Supplementary Figure 5. Predictive analysis of genes and signatures in patients with luminal A tumors (*N* = 49) for (A) DFS and (B) OS.** 13](#_Toc118824598)

[**Supplementary Figure 6. Prognostic analysis of genes and signatures in patients with luminal B tumors (*N* = 39) for (A) DFS and (B) OS.** 15](#_Toc118824599)

[**Supplementary Figure 7. Predictive analysis of genes and signatures in patients with luminal B tumors (*N* = 39) for (A) DFS and (B) OS.** 17](#_Toc118824600)

[**Supplementary Figure 8. Prognostic analysis of genes and signatures in patients with HER2-enriched tumors (*N* = 27) for (A) DFS and (B) OS.** 19](#_Toc118824601)

[**Supplementary Figure 9. Predictive analysis of genes and signatures in patients with HER2-enriched tumors (*N* = 27) for (A) DFS and (B) OS.** 21](#_Toc118824602)

[**Supplementary Figure 10. Prognostic analysis of genes and signatures according to treatment arm in patients with basal-like tumors (*N* = 26) for (A) DFS and (B) OS.** 23](#_Toc118824603)

[**Supplementary Figure 11. Predictive analysis of genes and signatures according to treatment arm in patients with basal-like tumors (*N* = 26) for (A) DFS and (B) OS.** 25](#_Toc118824604)

**Supplementary Table 1. Univariate and multivariate analyses of DFS and OS in patients with gene expression data (N = 141)**

|  | | **DFS** | | | | **OS** | | | |
| --- | --- | --- | --- | --- | --- | --- | --- | --- | --- |
|  |  | Univariate | | Multivariate | | Univariate | | Multivariate | |
|  |  | HR (95% CI) | *P* value | HR (95% CI) | *P* value | HR (95% CI) | *P* value | HR (95% CI) | *P* value |
| Arm | stCTX | 1 (ref.) |  | 1 (ref.) |  | 1 (ref.) |  | 1 (ref.) |  |
|  | ddCTX | 0.94  (0.59–1.49) | 0.790 | 0.78  (0.48–1.27) | 0.302 | 0.96  (0.60–1.54) | 0.857 | 0.80 (0.49–1.32) | 0.375 |
| Age | >43 years | 1 (ref.) |  | 1 (ref.) |  | 1 (ref.) |  | 1 (ref.) |  |
|  | ≤43 years | **2.13 (1.20–3.78)** | **0.010** | **2.95  (1.59–5.49)** | **0.001** | **2.12  (1.17–3.82)** | **0.013** | **3.69  (1.88–7.23)** | **<0.001** |
| pT | 1 | 1 (ref.) |  | 1 (ref.) |  | 1 (ref.) |  | 1 (ref.) |  |
|  | 2 | **2.21  (1.12–4.35)** | **0.021** | **2.53  (1.25–5.12)** | **0.010** | **3.20  (1.49–6.87)** | **0.003** | **4.29  (1.90–9.72)** | **<0.001** |
|  | 3 | **3.73  (1.77–7.88)** | **0.001** | **4.34  (1.91–9.88)** | **<0.001** | **4.54  (1.95–10.5)** | **<0.001** | **6.76  (2.64–17.3)** | **<0.001** |
|  | 4/X | **4.40  (1.61–12.0)** | **0.004** | **3.64  (1.21–10.9)** | **0.021** | **8.33  (2.99–23.2)** | **<0.001** | **7.82  (2.59–23.6)** | **<0.001** |
| Number of involved lymph nodes | 4–9 | 1 (ref.) |  | 1 (ref.) |  | **1 (ref.)** |  | 1 (ref.) |  |
|  | >9 | 1.65  (0.98-2.76) | 0.059 | 1.49  (0.85–2.63) | 0.165 | **1.87  (1.20–3.14)** | **0.017** | 1.64  (0.94–2.85) | 0.082 |
| Hormone receptor status | Positive | 1 (ref.) |  | 1 (ref.) |  | 1 (ref.) |  | 1 (ref.) |  |
|  | Negative | 1.06  (0.61–1.85) | 0.829 | 1.18  (0.63–2.21) | 0.609 | 0.88  (0.49–1.58) | 0.669 | 1.02  (0.53–1.97) | 0.943 |
| Type of local surgery | Mastectomy | 1 (ref.) |  | 1 (ref.) |  | 1 (ref.) |  | 1 (ref.) |  |
|  | Breast-conserving surgery | 0.66  (0.39-1.12) | 0.121 | 0.91  (0.52–1.59) | 0.734 | 0.94  (0.56­–1.55) | 0.800 | 1.47  (0.85–2.54) | 0.173 |

ddCTX, dose-dense chemotherapy; ref., reference; stCTX, standard dose chemotherapy.

Statistically significant associations are shown in bold.

**Supplementary Table 2. Multivariate analysis of prognostic associations for genes/signatures for DFS and OS in patients with HER2-enriched tumors (*N* = 27)**

| **Gene/ signature** | **DFS** | | | | **OS** | | | |
| --- | --- | --- | --- | --- | --- | --- | --- | --- |
|  | ddCTX | | stCTX | | ddCTX | | stCTX | |
|  | HR (95% CI)^a^ | *P* value | HR (95% CI)^a^ | *P* value | HR (95% CI)^a^ | *P* value | HR (95% CI)^a^ | *P* value |
| AR | NA | NS | NA | NS | **24.5 (1.12–533)** | **0.042** | NA | NS |
| TIGIT | NA | NS | 0.40 (0.15–1.07) | 0.069 | NA | NS | NA | NS |
| CD8+ T-cells | NA | NS | **0.27 (0.08–0.93)** | **0.039** | NA | NS | **0.09 (0.01–0.62)** | **0.015** |
| Cytotoxic cells | NA | NS | **0.35 (0.12–0.99)** | **0.049** | NA | NS | NA | NS |
| Mast cells | NA | NS | NA | NS | **11.0 (1.25–97.3)** | **0.031** | NA | NS |
| Treg | NA | NS | 0.34 (0.09–1.32) | 0.098 | NA | NS | 0.03 (0.0001–7.91) | 0.214 |

AR, androgen receptor; CI, confidence interval; ddCTX, dose-dense chemotherapy; DFS, disease-free survival; HR, hazard ratio; OS, overall survival; stCTX, standard-dosed chemotherapy; TIGIT, T-cell immunoreceptor with immunoglobulin and immunoreceptor tyrosine-based inhibition motif domains; Treg, regulatory T-cell.

Multivariate analysis was performed only for genes/signatures showing significant univariate associations. Statistically significant associations are shown in bold.

^a^ Adjusted for age (<43 versus ≥43 years), pT stage (T1 versus T2 versus T3 versus T4), and number of involved nodes (4–9 versus >9).

**Supplementary Table 3. Multivariate analysis of predictive associations for genes/signatures for DFS and OS in patients with HER2-enriched tumors (*N* = 27)**

| **Gene/signature** | **Category** | **DFS** | | | **OS** | | |
| --- | --- | --- | --- | --- | --- | --- | --- |
|  |  | HR (95% CI)^a^ | *P* value | *P* value (interaction) | HR (95% CI)^a^ | *P* value | *P* value (interaction) |
| Macrophages | <median | 1.05 (0.17–6.67) | 0.954 | 0.162 | 0.15 (0.02–1.35) | 0.090 | 0.212 |
|  | ≥median | 0.94 (0.13–6.67) | 0.955 |  | 0.99 (0.08–12.50) | 0.995 |  |
| Inflammatory chemokines | <median | 1.49 (0.13–16.67) | 0.744 | **0.004** | 1.08 (0.10–11.11) | 0.954 | **0.026** |
|  | ≥median | 0.46 (0.18–1.18) | 0.469 |  | 0.05 (0.00–1.54) | 0.085 |  |

CI, confidence interval; ddCTX, dose-dense chemotherapy; DFS, disease-free survival; HER2, human epidermal growth factor receptor-2; HR, hazard ratio; OS, overall survival; stCTX, standard-dosed CTX.

Statistically significant associations are shown in bold.

^a^ ddCTX versus stCTX, adjusted for age (<43 versus ≥43 years), pT stage (T1 versus T2 versus T3 versus T4), and number of involved nodes (4–9 versus >9). HR <1 favors ddCTX versus stCTX.

**Supplementary Table 4. Multivariate analysis of prognostic associations for genes and signatures for DFS and OS in patients with basal-like tumors (*N* = 26)**

| **Gene/ signature** | **DFS** | | | | **OS** | | | |
| --- | --- | --- | --- | --- | --- | --- | --- | --- |
|  | ddCTX | | stCTX | | ddCTX | | stCTX | |
|  | HR (95% CI)^a^ | *P* value | HR (95% CI)^a^ | *P* value | HR (95% CI)^a^ | *P* value | HR (95% CI)^a^ | *P* value |
| Luminal B | NA | NS | NA | NS | 0.001 (1.76E-9–605) | 0.310 | NA | NS |
| TIS | 0.42 (0.09–1.86) | 0.255 | 0.08 (0.005–1.45) | 0.086 | 0.27 (0.05–1.47) | 0.130 | NA | NS |
| ESR1 | **0.01 (1.52E-4–0.90)** | **0.045** | NA | NS | NA | NS | NA | NS |
| IDO1 | NA | NS | 0.19 (0.03–1.05) | 0.057 | 0.34 (0.10–1.11) | 0.073 | 0.42 (0.12–1.45) | 0.172 |
| PD-L1 | 0.34 (0.05–2.23) | 0.263 | 0.21 (0.03–1.59) | 0.131 | 0.001 (7.54E-7–2.24) | 0.081 | NA | NS |
| TIGIT | NA | NS | NA | NS | 0.21 (0.03–1.32) | 0.098 | NA | NS |
| CD8+ T-cells | NA | NS | NA | NS | 0.11 (0.01–11.49) | 0.096 | NA | NS |
| Cytotoxic cells | NA | NS | 0.31 (0.08–1.19) | 0.088 | 0.34 (0.07–1.57) | 0.166 | NA | NS |
| Cytotoxicity | 0.52 (0.12–2.27) | 0.384 | NA | NS | NA | NS | NA | NS |
| PD-1 | NA | NS | 0.05 (4.57E-4–5.68) | 0.192 | 0.17 (0.02–1.09) | 0.061 | NA | NS |

CI, confidence interval; ddCTX, dose-dense chemotherapy; DFS, disease-free survival; ESR1, oestrogen receptor-1; HR, hazard ratio; IDO1, indoleamine 2,3-dioxygenase-1; PD-1, programmed death-1; PD-L1, programmed death-ligand-1; OS, overall survival; stCTX, standard-dosed chemotherapy; TIGIT, T-cell immunoreceptor with immunoglobulin and immunoreceptor tyrosine-based inhibition motif domains; TIS, tumor inflammation signature.

Multivariate analysis was performed only for genes/signatures showing significant univariate associations. Statistically significant associations are shown in bold.

^a^ Adjusted for age (<43 versus ≥43 years), pT stage (T1 versus T2 versus T3 versus T4), and number of involved nodes (4–9 versus >9).

**Supplementary Figure 1. Adjusted survival curves for (A) DFS and (B) OS in patients with gene expression data (N = 141)**

**A**

**B**

ddCTX, dose-dense chemotherapy; DFS, disease-free survival; OS, overall survival; stCTX, standard dose chemotherapy.

**Supplementary Figure 2. Prognostic analysis of genes and signatures according to treatment arm in overall population (*N* = 141) for (A) DFS and (B) OS.**

**A**

APM, antigen processing machinery; AR, androgen receptor; BRCA, breast cancer susceptibility gene; CI, confidence interval; ddCTX, dose-dense chemotherapy; DFS, disease-free survival; ER, oestrogen receptor; ESR1, oestrogen receptor-1; FDR, false-discovery rate; FOXA1, forkhead box A1; HER2, human epidermal growth factor receptor-2; HR, hazard ratio; IDO1, indoleamine 2,3-dioxygenase-1; HRD, homologous recombination deficiency; IDO1, indoleamine 2,3-dioxygenase-1; OS, overall survival; PD-1, programmed death-1; PD-L1, programmed death-ligand-1; PD-L2, programmed death-ligand-2; PGR, progesterone receptor; ROR, risk of recurrence; stCTX, standard-dosed chemotherapy; TGF, transforming growth factor; TIGIT, T-cell immunoreceptor with immunoglobulin and immunoreceptor tyrosine-based inhibition motif domains; TIS, tumor inflammation signature; Treg, regulatory T-cell

Statistically significant associations are shown in bold.

**B**

APM, antigen processing machinery; AR, androgen receptor; BRCA, breast cancer susceptibility gene; CI, confidence interval; ddCTX, dose-dense chemotherapy; DFS, disease-free survival; ER, oestrogen receptor; ESR1, oestrogen receptor-1; FDR, false-discovery rate; FOXA1, forkhead box A1; HER2, human epidermal growth factor receptor-2; HR, hazard ratio; IDO1, indoleamine 2,3-dioxygenase-1; HRD, homologous recombination deficiency; IDO1, indoleamine 2,3-dioxygenase-1; OS, overall survival; PD-1, programmed death-1; PD-L1, programmed death-ligand-1; PD-L2, programmed death-ligand-2; PGR, progesterone receptor; ROR, risk of recurrence; stCTX, standard-dosed chemotherapy; TGF, transforming growth factor; TIGIT, T-cell immunoreceptor with immunoglobulin and immunoreceptor tyrosine-based inhibition motif domains; TIS, tumor inflammation signature; Treg, regulatory T-cell

Statistically significant associations are shown in bold.

**Supplementary Figure 3. Predictive analysis of genes and signatures in the overall population (*N* = 141) for (A) DFS and (B) OS.**

**A**

APM, antigen processing machinery; AR, androgen receptor; BRCA, breast cancer susceptibility gene; CI, confidence interval; ddCTX, dose-dense chemotherapy; DFS, disease-free survival; ER, ooestrogen receptor; ESR1, ooestrogen receptor-1; FDR, false-discovery rate; FOXA1, forkhead box A1; HER2, human epidermal growth factor receptor-2; HR, hazard ratio; IDO1, indoleamine 2,3-dioxygenase-1; HRD, homologous recombination deficiency; IDO1, indoleamine 2,3-dioxygenase-1; OS, overall survival; PD-1, programmed death-1; PD-L1, programmed death-ligand-1; PD-L2, programmed death-ligand-2; PGR, progesterone receptor; ROR, risk of recurrence; stCTX, standard-dosed chemotherapy; TGF, transforming growth factor; TIGIT, T-cell immunoreceptor with immunoglobulin and immunoreceptor tyrosine-based inhibition motif domains; TIS, tumor inflammation signature; Treg, regulatory T-cell

Statistically significant associations are shown in bold.

**B**

APM, antigen processing machinery; AR, androgen receptor; BRCA, breast cancer susceptibility gene; CI, confidence interval; ddCTX, dose-dense chemotherapy; DFS, disease-free survival; ER, oestrogen receptor; ESR1, oestrogen receptor-1; FDR, false-discovery rate; FOXA1, forkhead box A1; HER2, human epidermal growth factor receptor-2; HR, hazard ratio; IDO1, indoleamine 2,3-dioxygenase-1; HRD, homologous recombination deficiency; IDO1, indoleamine 2,3-dioxygenase-1; OS, overall survival; PD-1, programmed death-1; PD-L1, programmed death-ligand-1; PD-L2, programmed death-ligand-2; PGR, progesterone receptor; ROR, risk of recurrence; stCTX, standard-dosed chemotherapy; TGF, transforming growth factor; TIGIT, T-cell immunoreceptor with immunoglobulin and immunoreceptor tyrosine-based inhibition motif domains; TIS, tumor inflammation signature; Treg, regulatory T-cell

Statistically significant associations are shown in bold.

**Supplementary Figure 4. Prognostic analysis of genes and signatures in patients with luminal A tumors (*N* = 49) for (A) DFS and (B) OS.**

**A**

APM, antigen processing machinery; AR, androgen receptor; BRCA, breast cancer susceptibility gene; CI, confidence interval; ddCTX, dose-dense chemotherapy; DFS, disease-free survival; ER, oestrogen receptor; ESR1, oestrogen receptor-1; FDR, false-discovery rate; FOXA1, forkhead box A1; HER2, human epidermal growth factor receptor-2; HR, hazard ratio; IDO1, indoleamine 2,3-dioxygenase-1; HRD, homologous recombination deficiency; IDO1, indoleamine 2,3-dioxygenase-1; OS, overall survival; PD-1, programmed death-1; PD-L1, programmed death-ligand-1; PD-L2, programmed death-ligand-2; PGR, progesterone receptor; ROR, risk of recurrence; stCTX, standard-dosed chemotherapy; TGF, transforming growth factor; TIGIT, T-cell immunoreceptor with immunoglobulin and immunoreceptor tyrosine-based inhibition motif domains; TIS, tumor inflammation signature; Treg, regulatory T-cell

Statistically significant associations are shown in bold.

**B**

APM, antigen processing machinery; AR, androgen receptor; BRCA, breast cancer susceptibility gene; CI, confidence interval; ddCTX, dose-dense chemotherapy; DFS, disease-free survival; ER, oestrogen receptor; ESR1, oestrogen receptor-1; FDR, false-discovery rate; FOXA1, forkhead box A1; HER2, human epidermal growth factor receptor-2; HR, hazard ratio; IDO1, indoleamine 2,3-dioxygenase-1; HRD, homologous recombination deficiency; IDO1, indoleamine 2,3-dioxygenase-1; OS, overall survival; PD-1, programmed death-1; PD-L1, programmed death-ligand-1; PD-L2, programmed death-ligand-2; PGR, progesterone receptor; ROR, risk of recurrence; stCTX, standard-dosed chemotherapy; TGF, transforming growth factor; TIGIT, T-cell immunoreceptor with immunoglobulin and immunoreceptor tyrosine-based inhibition motif domains; TIS, tumor inflammation signature; Treg, regulatory T-cell

Statistically significant associations are shown in bold.

**Supplementary Figure 5. Predictive analysis of genes and signatures in patients with luminal A tumors (*N* = 49) for (A) DFS and (B) OS.**

**A**

APM, antigen processing machinery; AR, androgen receptor; BRCA, breast cancer susceptibility gene; CI, confidence interval; ddCTX, dose-dense chemotherapy; DFS, disease-free survival; ER, oestrogen receptor; ESR1, oestrogen receptor-1; FDR, false-discovery rate; FOXA1, forkhead box A1; HER2, human epidermal growth factor receptor-2; HR, hazard ratio; IDO1, indoleamine 2,3-dioxygenase-1; HRD, homologous recombination deficiency; IDO1, indoleamine 2,3-dioxygenase-1; OS, overall survival; PD-1, programmed death-1; PD-L1, programmed death-ligand-1; PD-L2, programmed death-ligand-2; PGR, progesterone receptor; ROR, risk of recurrence; stCTX, standard-dosed chemotherapy; TGF, transforming growth factor; TIGIT, T-cell immunoreceptor with immunoglobulin and immunoreceptor tyrosine-based inhibition motif domains; TIS, tumor inflammation signature; Treg, regulatory T-cell

Statistically significant associations are shown in bold.

**B**

APM, antigen processing machinery; AR, androgen receptor; BRCA, breast cancer susceptibility gene; CI, confidence interval; ddCTX, dose-dense chemotherapy; DFS, disease-free survival; ER, oestrogen receptor; ESR1, oestrogen receptor-1; FDR, false-discovery rate; FOXA1, forkhead box A1; HER2, human epidermal growth factor receptor-2; HR, hazard ratio; IDO1, indoleamine 2,3-dioxygenase-1; HRD, homologous recombination deficiency; IDO1, indoleamine 2,3-dioxygenase-1; OS, overall survival; PD-1, programmed death-1; PD-L1, programmed death-ligand-1; PD-L2, programmed death-ligand-2; PGR, progesterone receptor; ROR, risk of recurrence; stCTX, standard-dosed chemotherapy; TGF, transforming growth factor; TIGIT, T-cell immunoreceptor with immunoglobulin and immunoreceptor tyrosine-based inhibition motif domains; TIS, tumor inflammation signature; Treg, regulatory T-cell

Statistically significant associations are shown in bold.

**Supplementary Figure 6. Prognostic analysis of genes and signatures in patients with luminal B tumors (*N* = 39) for (A) DFS and (B) OS.**

**A**

APM, antigen processing machinery; AR, androgen receptor; BRCA, breast cancer susceptibility gene; CI, confidence interval; ddCTX, dose-dense chemotherapy; DFS, disease-free survival; ER, oestrogen receptor; ESR1, oestrogen receptor-1; FDR, false-discovery rate; FOXA1, forkhead box A1; HER2, human epidermal growth factor receptor-2; HR, hazard ratio; IDO1, indoleamine 2,3-dioxygenase-1; HRD, homologous recombination deficiency; IDO1, indoleamine 2,3-dioxygenase-1; OS, overall survival; PD-1, programmed death-1; PD-L1, programmed death-ligand-1; PD-L2, programmed death-ligand-2; PGR, progesterone receptor; ROR, risk of recurrence; stCTX, standard-dosed chemotherapy; TGF, transforming growth factor; TIGIT, T-cell immunoreceptor with immunoglobulin and immunoreceptor tyrosine-based inhibition motif domains; TIS, tumor inflammation signature; Treg, regulatory T-cell

Statistically significant associations are shown in bold.

**B**

APM, antigen processing machinery; AR, androgen receptor; BRCA, breast cancer susceptibility gene; CI, confidence interval; ddCTX, dose-dense chemotherapy; DFS, disease-free survival; ER, oestrogen receptor; ESR1, oestrogen receptor-1; FDR, false-discovery rate; FOXA1, forkhead box A1; HER2, human epidermal growth factor receptor-2; HR, hazard ratio; IDO1, indoleamine 2,3-dioxygenase-1; HRD, homologous recombination deficiency; IDO1, indoleamine 2,3-dioxygenase-1; OS, overall survival; PD-1, programmed death-1; PD-L1, programmed death-ligand-1; PD-L2, programmed death-ligand-2; PGR, progesterone receptor; ROR, risk of recurrence; stCTX, standard-dosed chemotherapy; TGF, transforming growth factor; TIGIT, T-cell immunoreceptor with immunoglobulin and immunoreceptor tyrosine-based inhibition motif domains; TIS, tumor inflammation signature; Treg, regulatory T-cell

Statistically significant associations are shown in bold.

**Supplementary Figure 7. Predictive analysis of genes and signatures in patients with luminal B tumors (*N* = 39) for (A) DFS and (B) OS.**

**A**

APM, antigen processing machinery; AR, androgen receptor; BRCA, breast cancer susceptibility gene; CI, confidence interval; ddCTX, dose-dense chemotherapy; DFS, disease-free survival; ER, oestrogen receptor; ESR1, oestrogen receptor-1; FDR, false-discovery rate; FOXA1, forkhead box A1; HER2, human epidermal growth factor receptor-2; HR, hazard ratio; IDO1, indoleamine 2,3-dioxygenase-1; HRD, homologous recombination deficiency; IDO1, indoleamine 2,3-dioxygenase-1; OS, overall survival; PD-1, programmed death-1; PD-L1, programmed death-ligand-1; PD-L2, programmed death-ligand-2; PGR, progesterone receptor; ROR, risk of recurrence; stCTX, standard-dosed chemotherapy; TGF, transforming growth factor; TIGIT, T-cell immunoreceptor with immunoglobulin and immunoreceptor tyrosine-based inhibition motif domains; TIS, tumor inflammation signature; Treg, regulatory T-cell

Statistically significant associations are shown in bold.

**B**

APM, antigen processing machinery; AR, androgen receptor; BRCA, breast cancer susceptibility gene; CI, confidence interval; ddCTX, dose-dense chemotherapy; DFS, disease-free survival; ER, oestrogen receptor; ESR1, oestrogen receptor-1; FDR, false-discovery rate; FOXA1, forkhead box A1; HER2, human epidermal growth factor receptor-2; HR, hazard ratio; IDO1, indoleamine 2,3-dioxygenase-1; HRD, homologous recombination deficiency; IDO1, indoleamine 2,3-dioxygenase-1; OS, overall survival; PD-1, programmed death-1; PD-L1, programmed death-ligand-1; PD-L2, programmed death-ligand-2; PGR, progesterone receptor; ROR, risk of recurrence; stCTX, standard-dosed chemotherapy; TGF, transforming growth factor; TIGIT, T-cell immunoreceptor with immunoglobulin and immunoreceptor tyrosine-based inhibition motif domains; TIS, tumor inflammation signature; Treg, regulatory T-cell

Statistically significant associations are shown in bold.

**Supplementary Figure 8. Prognostic analysis of genes and signatures in patients with HER2-enriched tumors (*N* = 27) for (A) DFS and (B) OS.**

**A**

APM, antigen processing machinery; AR, androgen receptor; BRCA, breast cancer susceptibility gene; CI, confidence interval; ddCTX, dose-dense chemotherapy; DFS, disease-free survival; ER, oestrogen receptor; ESR1, oestrogen receptor-1; FDR, false-discovery rate; FOXA1, forkhead box A1; HER2, human epidermal growth factor receptor-2; HR, hazard ratio; IDO1, indoleamine 2,3-dioxygenase-1; HRD, homologous recombination deficiency; IDO1, indoleamine 2,3-dioxygenase-1; OS, overall survival; PD-1, programmed death-1; PD-L1, programmed death-ligand-1; PD-L2, programmed death-ligand-2; PGR, progesterone receptor; ROR, risk of recurrence; stCTX, standard-dosed chemotherapy; TGF, transforming growth factor; TIGIT, T-cell immunoreceptor with immunoglobulin and immunoreceptor tyrosine-based inhibition motif domains; TIS, tumor inflammation signature; Treg, regulatory T-cell

Statistically significant associations are shown in bold.

**B**

APM, antigen processing machinery; AR, androgen receptor; BRCA, breast cancer susceptibility gene; CI, confidence interval; ddCTX, dose-dense chemotherapy; DFS, disease-free survival; ER, oestrogen receptor; ESR1, oestrogen receptor-1; FDR, false-discovery rate; FOXA1, forkhead box A1; HER2, human epidermal growth factor receptor-2; HR, hazard ratio; IDO1, indoleamine 2,3-dioxygenase-1; HRD, homologous recombination deficiency; IDO1, indoleamine 2,3-dioxygenase-1; OS, overall survival; PD-1, programmed death-1; PD-L1, programmed death-ligand-1; PD-L2, programmed death-ligand-2; PGR, progesterone receptor; ROR, risk of recurrence; stCTX, standard-dosed chemotherapy; TGF, transforming growth factor; TIGIT, T-cell immunoreceptor with immunoglobulin and immunoreceptor tyrosine-based inhibition motif domains; TIS, tumor inflammation signature; Treg, regulatory T-cell

Statistically significant associations are shown in bold.

**Supplementary Figure 9. Predictive analysis of genes and signatures in patients with HER2-enriched tumors (*N* = 27) for (A) DFS and (B) OS.**

**A**

APM, antigen processing machinery; AR, androgen receptor; BRCA, breast cancer susceptibility gene; CI, confidence interval; ddCTX, dose-dense chemotherapy; DFS, disease-free survival; ER, oestrogen receptor; ESR1, oestrogen receptor-1; FDR, false-discovery rate; FOXA1, forkhead box A1; HER2, human epidermal growth factor receptor-2; HR, hazard ratio; IDO1, indoleamine 2,3-dioxygenase-1; HRD, homologous recombination deficiency; IDO1, indoleamine 2,3-dioxygenase-1; OS, overall survival; PD-1, programmed death-1; PD-L1, programmed death-ligand-1; PD-L2, programmed death-ligand-2; PGR, progesterone receptor; ROR, risk of recurrence; stCTX, standard-dosed chemotherapy; TGF, transforming growth factor; TIGIT, T-cell immunoreceptor with immunoglobulin and immunoreceptor tyrosine-based inhibition motif domains; TIS, tumor inflammation signature; Treg, regulatory T-cell

Statistically significant associations are shown in bold.

**B**

APM, antigen processing machinery; AR, androgen receptor; BRCA, breast cancer susceptibility gene; CI, confidence interval; ddCTX, dose-dense chemotherapy; DFS, disease-free survival; ER, oestrogen receptor; ESR1, oestrogen receptor-1; FDR, false-discovery rate; FOXA1, forkhead box A1; HER2, human epidermal growth factor receptor-2; HR, hazard ratio; IDO1, indoleamine 2,3-dioxygenase-1; HRD, homologous recombination deficiency; IDO1, indoleamine 2,3-dioxygenase-1; OS, overall survival; PD-1, programmed death-1; PD-L1, programmed death-ligand-1; PD-L2, programmed death-ligand-2; PGR, progesterone receptor; ROR, risk of recurrence; stCTX, standard-dosed chemotherapy; TGF, transforming growth factor; TIGIT, T-cell immunoreceptor with immunoglobulin and immunoreceptor tyrosine-based inhibition motif domains; TIS, tumor inflammation signature; Treg, regulatory T-cell

Statistically significant associations are shown in bold.

**Supplementary Figure 10. Prognostic analysis of genes and signatures according to treatment arm in patients with basal-like tumors (*N* = 26) for (A) DFS and (B) OS.**

**A**

APM, antigen processing machinery; AR, androgen receptor; BRCA, breast cancer susceptibility gene; CI, confidence interval; ddCTX, dose-dense chemotherapy; DFS, disease-free survival; ER, oestrogen receptor; ESR1, oestrogen receptor-1; FDR, false-discovery rate; FOXA1, forkhead box A1; HER2, human epidermal growth factor receptor-2; HR, hazard ratio; IDO1, indoleamine 2,3-dioxygenase-1; HRD, homologous recombination deficiency; IDO1, indoleamine 2,3-dioxygenase-1; OS, overall survival; PD-1, programmed death-1; PD-L1, programmed death-ligand-1; PD-L2, programmed death-ligand-2; PGR, progesterone receptor; ROR, risk of recurrence; stCTX, standard-dosed chemotherapy; TGF, transforming growth factor; TIGIT, T-cell immunoreceptor with immunoglobulin and immunoreceptor tyrosine-based inhibition motif domains; TIS, tumor inflammation signature; Treg, regulatory T-cell

Statistically significant associations are shown in bold.

**B**

APM, antigen processing machinery; AR, androgen receptor; BRCA, breast cancer susceptibility gene; CI, confidence interval; ddCTX, dose-dense chemotherapy; DFS, disease-free survival; ER, oestrogen receptor; ESR1, oestrogen receptor-1; FDR, false-discovery rate; FOXA1, forkhead box A1; HER2, human epidermal growth factor receptor-2; HR, hazard ratio; IDO1, indoleamine 2,3-dioxygenase-1; HRD, homologous recombination deficiency; IDO1, indoleamine 2,3-dioxygenase-1; OS, overall survival; PD-1, programmed death-1; PD-L1, programmed death-ligand-1; PD-L2, programmed death-ligand-2; PGR, progesterone receptor; ROR, risk of recurrence; stCTX, standard-dosed chemotherapy; TGF, transforming growth factor; TIGIT, T-cell immunoreceptor with immunoglobulin and immunoreceptor tyrosine-based inhibition motif domains; TIS, tumor inflammation signature; Treg, regulatory T-cell

Statistically significant associations are shown in bold.

**Supplementary Figure 11. Predictive analysis of genes and signatures according to treatment arm in patients with basal-like tumors (*N* = 26) for (A) DFS and (B) OS.**

**A**

APM, antigen processing machinery; AR, androgen receptor; BRCA, breast cancer susceptibility gene; CI, confidence interval; ddCTX, dose-dense chemotherapy; DFS, disease-free survival; ER, oestrogen receptor; ESR1, oestrogen receptor-1; FDR, false-discovery rate; FOXA1, forkhead box A1; HER2, human epidermal growth factor receptor-2; HR, hazard ratio; IDO1, indoleamine 2,3-dioxygenase-1; HRD, homologous recombination deficiency; IDO1, indoleamine 2,3-dioxygenase-1; OS, overall survival; PD-1, programmed death-1; PD-L1, programmed death-ligand-1; PD-L2, programmed death-ligand-2; PGR, progesterone receptor; ROR, risk of recurrence; stCTX, standard-dosed chemotherapy; TGF, transforming growth factor; TIGIT, T-cell immunoreceptor with immunoglobulin and immunoreceptor tyrosine-based inhibition motif domains; TIS, tumor inflammation signature; Treg, regulatory T-cell

Statistically significant associations are shown in bold.

**B**

APM, antigen processing machinery; AR, androgen receptor; BRCA, breast cancer susceptibility gene; CI, confidence interval; ddCTX, dose-dense chemotherapy; DFS, disease-free survival; ER, oestrogen receptor; ESR1, oestrogen receptor-1; FDR, false-discovery rate; FOXA1, forkhead box A1; HER2, human epidermal growth factor receptor-2; HR, hazard ratio; IDO1, indoleamine 2,3-dioxygenase-1; HRD, homologous recombination deficiency; IDO1, indoleamine 2,3-dioxygenase-1; OS, overall survival; PD-1, programmed death-1; PD-L1, programmed death-ligand-1; PD-L2, programmed death-ligand-2; PGR, progesterone receptor; ROR, risk of recurrence; stCTX, standard-dosed chemotherapy; TGF, transforming growth factor; TIGIT, T-cell immunoreceptor with immunoglobulin and immunoreceptor tyrosine-based inhibition motif domains; TIS, tumor inflammation signature; Treg, regulatory T-cell

Statistically significant associations are shown in bold.
